# Piwi Is Required in Multiple Cell Types to Control Germline Stem Cell Lineage Development in the *Drosophila* Ovary

**DOI:** 10.1371/journal.pone.0090267

**Published:** 2014-03-21

**Authors:** Xing Ma, Su Wang, Trieu Do, Xiaoqing Song, Mayu Inaba, Yoshiya Nishimoto, Lu-ping Liu, Yuan Gao, Ying Mao, Hui Li, William McDowell, Jungeun Park, Kate Malanowski, Allison Peak, Anoja Perera, Hua Li, Karin Gaudenz, Jeff Haug, Yukiko Yamashita, Haifan Lin, Jian-quan Ni, Ting Xie

**Affiliations:** 1 Stowers Institute for Medical Research, Kansas City, Missouri, United States of America; 2 Department of Anatomy and Cell Biology, University of Kansas School of Medicine, Kansas City, Kansas, United States of America; 3 Life Sciences Institute, Center for Stem Cell Biology, University of Michigan, Ann Arbor, Michigan, United States of America; 4 School of Medicine, Tsinghua University, Beijing, China; 5 Yale Stem Cell Center, Yale University School of Medicine, New Haven, Connecticut, United Sates of America; Technische Universität Dresden, Germany

## Abstract

The piRNA pathway plays an important role in maintaining genome stability in the germ line by silencing transposable elements (TEs) from fly to mammals. As a highly conserved piRNA pathway component, Piwi is widely expressed in both germ cells and somatic cells in the *Drosophila* ovary and is required for piRNA production in both cell types. In addition to its known role in somatic cap cells to maintain germline stem cells (GSCs), this study has demonstrated that Piwi has novel functions in somatic cells and germ cells of the *Drosophila* ovary to promote germ cell differentiation. *Piwi* knockdown in escort cells causes a reduction in escort cell (EC) number and accumulation of undifferentiated germ cells, some of which show active BMP signaling, indicating that Piwi is required to maintain ECs and promote germ cell differentiation. Simultaneous knockdown of *dpp*, encoding a BMP, in ECs can partially rescue the germ cell differentiation defect, indicating that Piwi is required in ECs to repress *dpp*. Consistent with its key role in piRNA production, TE transcripts increase significantly and DNA damage is also elevated in the *piwi* knockdown somatic cells. Germ cell-specific knockdown of *piwi* surprisingly causes depletion of germ cells before adulthood, suggesting that Piwi might control primordial germ cell maintenance or GSC establishment. Finally, Piwi inactivation in the germ line of the adult ovary leads to gradual GSC loss and germ cell differentiation defects, indicating the intrinsic role of Piwi in adult GSC maintenance and differentiation. This study has revealed new germline requirement of Piwi in controlling GSC maintenance and lineage differentiation as well as its new somatic function in promoting germ cell differentiation. Therefore, Piwi is required in multiple cell types to control GSC lineage development in the *Drosophila* ovary.

## Introduction

Small RNAs have received much attention in recent years because of their important and diverse roles in the regulation of various biological processes [1], [2], [3],[4],[5]. In contrast to other small RNAs, Piwi-associated small RNAs, also known as piRNAs, are abundantly expressed in germ cells of organisms ranging from *C. elegans* to human, and have emerged as an important class of small RNAs for maintaining genome stability in germ cells [6], [7], [8], [9]. Recent studies have shown that piRNAs also function in somatic cells to regulate gene expression and repress TEs [10], [11], [12], [13], [14], [15]. However, biological functions of piRNAs still remain poorly defined.

**Figure 1:**
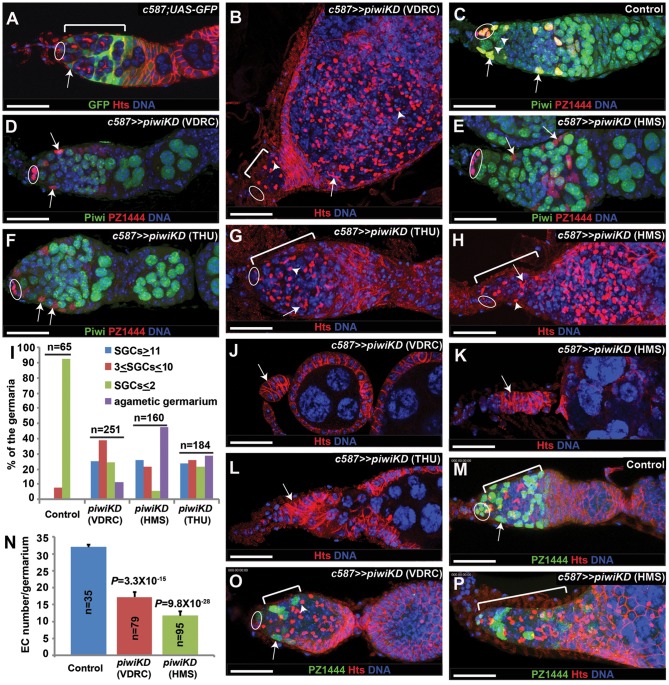
Piwi is required in ECs to promote germ cell differentiation and maintain EC survival. Ovals indicate cap cells, whereas brackets denote the germarial region covered by ECs. (**A**) *c587* drives GFP expression specifically in ECs (arrow). (**B**) *c587*-mediated *piwi* knockdown (*piwiKD*) leads to an accumulation of many single germ cells (SGCs) carrying a spectrosome (arrowhead) mixed with differentiated cysts containing a branched fusome (arrow) in the germarium and its associated egg chamber. (**C**) Piwi protein is expressed in *PZ1444*-positive cap cells and ECs (arrow) as well as in follicle cells and germ cells. (**D**–**F**) *c587*-mediated *piwiKD* by three independent RNAi lines efficiently eliminates Piwi protein expression in *PZ1444*-positive cap cells and ECs (arrows), whereas Piwi protein expression in germ cells remains normal. (**G**–**I**) *c587*-mediated *piwiKD* by THU and HMS lines leads to the accumulation of SGCs (arrowhead) mixed with differentiated cysts (arrow). **I** shows the quantitative results on the numbers of SGCs and agametic germaria (n indicates total germaria examined). (**J**–**L**) *c587*-mediated *piwiKD* causes the formation of the germaria (arrows) containing no germ cells. (**M**–**P**) *c587*-mediated *piwiKD* (**O**, **P**) results in a significant reduction in EC numbers in comparison with the control (**M**). **N** shows the quantitative results on EC numbers (n indicates total germaria examined). Scale bars: 25 µm.

The *Drosophila* ovary is an attractive system for studying stem cell lineage development [16]. Two types of stem cells, germline stem cells (GSCs) and follicular stem cells (FSCs), are responsible for continuously producing differentiated germ cell cysts and follicle cells, respectively, which are assembled into egg chambers that eventually develop into mature oocytes. Two or three GSCs are situated in the tip of each ovariole, known as the germarium, and can be easily identified by their direct contact with cap cells and presence of an anteriorly localized spectrosome (Fig. 1A). Immediate GSC daughters, also known as cystoblasts (CBs), move away from cap cells and undergo four rounds of synchronized cell division to form 2-cell, 4-cell, 8-cell and 16-cell cysts. CBs and cysts are tightly encased by cellular processes of escort cells (ECs), also known as inner germarial sheath cells (Fig. 1A). Genetic and cell biological studies have shown that terminal filament (TF)/cap cells and anterior ECs form the self-renewing niche for GSCs, which provides the essential BMP signal for repressing GSC differentiation and thereby maintaining their self-renewal [16].

Based on recent studies from us and others [17], [18], we have recently proposed that posterior ECs function as the microenvironment or niche for promoting germ cell differentiation [18]. One of the key functions of ECs is to prevent BMP signaling via two distinct strategies. First, EGFR-MAPK signaling has been proposed to directly repress expression of *dally*, encoding a proteoglycan facilitating BMP signal transduction and diffusion [17]. Rho signaling and Eggless have been shown to repress *dally* expression in ECs, thus promoting germ cell differentiation, but it remains unclear how they might regulate *dally* expression [18], [19]. The second strategy is direct repression of transcription of *dpp*, which encodes a BMP ligand essential for GSC self-renewal in *Drosophila*. Histone lysine-specific demethylase 1 (Lsd1, a chromatin regulator) and Rho signaling have been shown to repress *dpp* transcription in ECs [18], [20]. *dpp* knockdown can partially rescue the germ cell differentiation defects caused by inactivation of Lsd1 and Rho signaling in ECs, indicating that *dpp* upregulation contributes to the germ cell differentiation defects. Therefore, ECs have so far been demonstrated to promote germ cell differentiation by preventing the spreading of BMP signaling.

It is the *Drosophila* ovary in which the first piRNA regulator, *piwi*, was identified for its critical role in maintaining GSCs [21], [22]. Although it is expressed in all germ cells and somatic cells of the *Drosophila* ovary, it has been suggested to function in TF/cap cells for maintaining GSCs [22], [23]. In addition, Piwi is also required intrinsically to promote GSC division and primordial germ cell formation [24], [25]. In *Drosophila* ovarian somatic cells, Yb works with Piwi to control primary piRNA biogenesis [12], [14], [26], and is also suggested to work in TF/cap cells to maintain GSC self-renewal [27]. In addition, recent studies have shown that Armitage (Armi), Vreteno (Vret) and Tdrd12 are also required in somatic cells to control primary piRNA biogenesis [28], [29], [30]. Inactivation of histone H3K9 trimethylase Eggless function in ECs leads to defective piRNA biogenesis, upregulation of transposons and germ cell differentiation defects, indicating that piRNAs are important for maintaining EC function by repressing transposons [19], [31]. Consistently, *vret* mutants also have a germ cell differentiation defect, which can be rescued by somatic cell-specific expression of *vret* [28], [29]. In this study, we show that *piwi* is required in ECs and germline to control germ cell differentiation.

## Results

### Piwi is required in ECs to control germ cell differentiation and EC survival

To identify the genes that are required in ECs for controlling germ cell differentiation, we carried out a genetic screen using an EC-expressing *gal4* driver *c587* and transgenic *UAS-RNAi* lines from the Vienna *Drosophila* RNAi Center (VDRC). These VDRC RNAi transgenic lines were designed based on the production of a long double-stranded RNA structure that can be further processed into small double-stranded RNAs degrading target mRNAs, and have been used to carry out genetic screens in various *Drosophila* tissue types [32], [33], [34]. *c587* is expressed specifically in ECs and early follicle cell progenitors based on the expression of *UAS-GFP* [35](Fig. 1A). In our screen, *piwi* was identified for its requirement in ECs to control germ cell differentiation as *c587*-mediated knockdown of *piwi* (*piwiKD*) causes the accumulation of spectrosome-containing ill-differentiated single germ cells (SGCs) located distantly from cap cells in the knockdown germaria, which is in great contrast with the control germaria (Fig. 1B). Although GSCs and cystoblasts (CBs, immediate GSC progeny) contain a spherical spectrosome, only GSCs directly contact cap cells. Differentiated 2-cell, 4-cell, 8-cell and 16-cell cysts contain a branched fusome, and can be easily distinguished from GSCs and CBs. The spectrosome and fusome are the same germ cell-specific intracellular organelle with different morphologies, which can be reliably labeled with antibodies against their components such as Hu li-tai shao (Hts) [36] (Fig. 1A and 1B). To verify the RNAi-mediated Piwi knockdown efficiency, we used polyclonal anti-Piwi antibodies to examine Piwi protein expression in the control and *piwiKD* germaria, in which cap cells and ECs are marked by the *PZ1444* enhancer trap line [37]. Two additional microRNA-based *UAS-RNAi* transgenic strains were also used in this study: one RNAi strain was generated by the Perrimon Laboratory at Harvard Medical School, HMS [38], [39], and the other RNAi line, THU, was generated to target a different *piwi* sequence by the Ni laboratory at Tsinghua University. The PZ1444 enhancer trap line expresses nuclear β-galactosidase protein in cap cells and ECs, which can be reliably distinguished by their location and morphology [37], [40](Fig. 1C). As previously reported [23], [41], Piwi is generally expressed in both somatic cells and germ cells of the control germaria, but ECs express higher levels of Piwi than cap cells (Fig. 1C). Indeed, all the RNAi lines can efficiently eliminate Piwi expression in PZ1444-positive ECs and cap cells, while Piwi expression in germ cells including GSCs remains unchanged in the *piwiKD* germaria (Fig. 1D–1F). *c587*-mediated expression of the HMS and THU RNAi lines can also cause the accumulation of ill-differentiated SGCs outside the GSC niche, similar to the VDRC line (Fig. 1G and 1H). Because a wild-type germarium normally contains one or two CBs, a germarium containing three or more SGCs is considered to exhibit the germ cell differentiation defect [42]. To determine the severity of the germ cell differentiation defects, we classified the *piwi* knockdown germaria into three categories: normal (SGCs≤2), moderate differentiation defect (3≤SGCs≤10) and severe differentiation defect (SGCs≥11). Among the germaria in which *piwi* is knocked down in ECs by three independent RNAi lines, 40–60% of them have 3 or more SGCs, and approximately 20% of them have 11 or more SGCs (Fig. 1I). In addition, egg chambers are also often filled with undifferentiated spectrosome-containing SGCs (Fig. 1B and 1H). Quantitatively, the three RNAi lines produce similar degrees of germ cell differentiation defects (Fig. 1I). As shown in Fig. 1I, there are some variations on SGC numbers in the knockdown germaria by different RNAi strains, and Fig. 1B, 1G and 1H reflect the variations among the three RNAi strains. In addition, we have observed that 10–50% of the *piwiKD* germaria by the three independent RNAi lines completely lose GSCs and become agametic, suggesting that Piwi is required in somatic cells for maintaining GSCs (Fig. 1I–L). These results indicate that Piwi is indeed required in ECs to promote germ cell differentiation and in ECs, cap cells or both to maintain GSCs.

The germarial region of the *piwiKD* ovaries appears to be reduced in size, suggesting that the EC number may also be reduced as well (Fig. 1B and 1H). Our previous studies have suggested that the severity of EC loss is positively correlated with the severity of the germ cell differentiation defects [18], [19]. We then quantified EC numbers in the control and *piwiKD* germaria. In contrast with the control germarium containing an average of 35 ECs (Fig. 1M and 1N), the *piwiKD* germarium contains significantly fewer ECs (Fig. 1N–P). Because ECs rarely proliferate, the reduction in EC number could be due to apoptosis. To directly test this idea, we used TUNEL labeling to detect dying ECs identified by PZ1444 expression. Indeed, there is a consistent increase in apoptotic *piwiKD* ECs by the three independent RNAi lines (Fig. S1). These results demonstrate that Piwi is also required for maintaining EC survival.

### Piwi functions in adult ECs to promote germ cell differentiation

**Figure 2:**
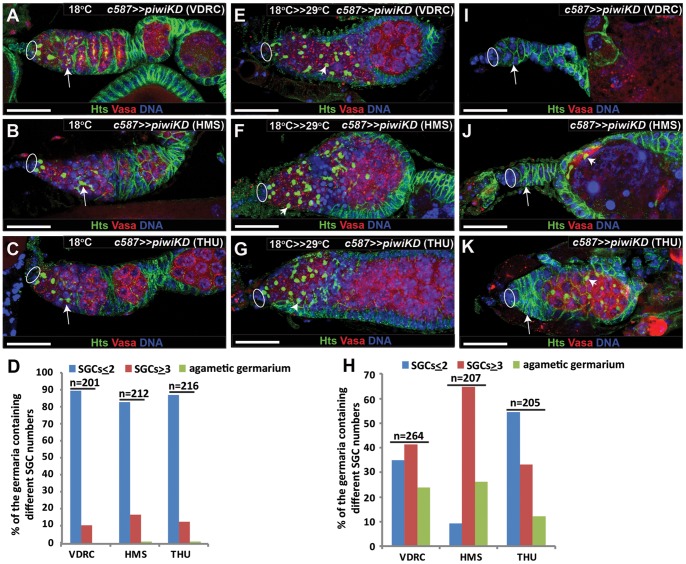
Piwi is required in adult ECs to maintain GSCs and promote germ cell differentiation. Ovals, arrows and arrowheads indicate cap cells, branched fusomes and spherical spectrosomes, respectively. Germaria in **A**–**C**, **E**–**G** and **I**–**K** are labeled for Hts (green, spectrosome/fusome), Vasa (red, germ cells) and DNA (blue). (**A**–**D**) At 18°C, *c587*-mediated *piwiKD* does not affect GSC and SGC numbers due to low RNAi expression. **D** represents quantitative results on the numbers of SGCs and germless germaria. (**E**–**H**) 1w after shifting to 29°C, *c587*-mediated *piwiKD* leads to an accumulation of excess SGCs in the germaria. **H** represents quantitative results on the numbers of SGCs and germless germaria. (**I**–**K**) 1w after shifting to 29°C, *c587*-mediated *piwiKD* causes some germaria to completely lose germ cells including GSCs. Scale bars: 25 µm.

Since *c587* is known to be expressed by most, if not all, somatic precursor cells in the female gonad [43], the differentiation defects and the GSC loss phenotype caused by *piwi* knockdown could be due to its early requirement in somatic gonadal precursors. To definitively determine if Piwi is required in adult ECs to control germ cell differentiation, we carried out temperature shift experiments to inactivate Piwi function specifically in adult ECs. When the genetic crosses were carried out at 18°C, which lowers *piwi* RNAi expression and thus its knockdown effect, the germaria show almost normal germ cell differentiation and GSC maintenance because all the *piwiKD* germaria have normal SGC numbers and still retain two or three GSCs (Fig. 2A–D). After the adult females emerged at 18°C, they were cultured at 29°C for a week to increase RNAi expression and *piwi* knockdown efficiency and thus inactivate Piwi function in adult ECs. Interestingly, the number of the germaria carrying three or more SGCs drastically increases, indicating that Piwi is indeed required in adult ECs to promote germ cell differentiation (Fig. 2E–H). Similarly, the numbers of the germaria containing no GSCs also increase following Piwi knockdown by the three RNAi lines (Fig. 2H–K). In addition, Piwi expression is still reduced in cap cells, suggesting that *c587* is likely expressed at low levels in cap cells (Fig. S2). Since the previous findings have shown that ECs also contribute to GSC maintenance [18], [19], [44], the GSC loss phenotype could come either from Piwi knockdown in cap cells, ECs or both. Taken together, these results indicate that Piwi is required in adult ECs to promote germ cell differentiation as well as in adult ECs, adult cap cells or both for GSC maintenance.

### Piwi is required in ECs to prevent BMP signaling in differentiated germ cells

**Figure 3:**
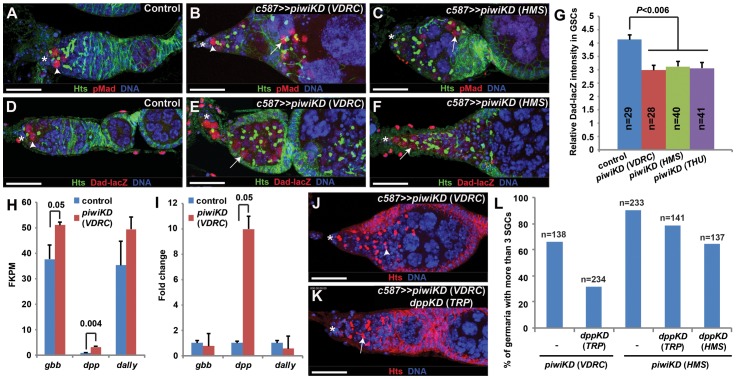
Piwi knockdown in ECs results in an elevation of BMP signaling in SGCs outside the GSC niche. Asterisks indicate the GSC niche. (**A**–**C**) Some SGCs (arrows) outside the GSC niche are positive for pMad in the *piwiKD* germaria (**B**, **C**) in addition to GSCs (arrowhead) in contrast with the control germarium in which only GSCs (arrowhead) are positive (**A**). (**D**–**G**) Some SGCs (arrows) outside the GSC niche are positive for *Dad-lacZ* in the *piwiKD* germaria (**E**, **F**) in contrast with the control germarium in which only GSCs (arrowhead) are positive (**D**). **G** shows quantification results on *Dad-lacZ* expression in GSCs. (**H**, **I**) RNA sequencing (**H**) and qRT-PCR (**I**) results show that mRNA expression levels for *dpp*, but not for *gbb* and *dally*, are significantly upregulated in the *piwi* knockdown ECs compared to the control ECs (FKPM stands for fragments per kilobase of exon per millions of reads). (**J**–**L**) *c587*-mediated *dpp* knockdown can partially rescue the germ cell differentiation defects caused by *piwi* knockdown. **L** shows quantification results on percentages of germaria carrying three or more SGCs among the *piwiKD* and *piwi dppKD* germaria, which still contain at least one GSC. Scale bars: 25 µm.

Previous studies have shown that the germ cell differentiation defects caused by defective EC function result from elevated BMP signaling [17], [18], [19]. To determine if BMP signaling activity is augmented in the germ cells of the *piwiKD* germaria, we examined the expression of pMad, *Dad-lacZ* and *bam-GFP*, three BMP signaling activity reporters in *Drosophila*, in the control and *piwiKD* germaria. Activation of BMP receptors (Tkv and Sax) upon BMP ligand binding leads to production of phosphorylated Mad (pMad), which translocates into the nucleus with Medea, a SMAD4 homolog, to activate *Dad* expression and repress *bam* expression in GSCs [35], [45], [46]. In contrast with the control germarium in which pMad accumulates primarily in GSCs (Fig. 3A), pMad is also expressed in some, but not all, SGCs outside the GSC niche of the *piwiKD* germaria, indicating that BMP signaling activity indeed spreads outside the GSC niche (Fig. 3B, 3C and S3A). *bam-GFP* and *Dad-lacZ* can recapitulate the expression patterns of *bam* and *Dad* in the control germarium: *bam-GFP* is normally expressed in differentiated germ cells but is absent from GSCs [47], while *Dad-lacZ* is normally expressed in GSCs but is absent in differentiated germ cells [35], [45], [46] (Fig. 3D and Fig. S3C). Although it is still expressed in GSCs of the *piwiKD* germaria (Fig. 3E, 3F and S3B), *Dad-lacZ* reduces its expression by about 25% based on quantification results (Fig. 3G). Although *bam-GFP* remains repressed in the GSCs of the *piwiKD* germaria, it fails to be upregulated in some SGCs outside the GSC niche in the *piwiKD* germaria as in control CBs (Fig. S3C–F). These results indicate that Piwi is required in ECs to prevent BMP signaling activity in differentiated germ cells.

Previous studies have revealed that the elevated transcription of *dpp*, which encodes a BMP ligand, in ECs can contribute to increased BMP signaling in differentiated germ cells [18], [19], [20]. In *Drosophila*, another BMP-encoding gene, *gbb*, is also expressed in the germarium and is required for maintaining GSCs [35]. In addition, *dally* upregulation in ECs has also been shown to be responsible for BMP signaling activity elevation [17], [48], [49]. To determine if the elevated BMP signaling activity in SGCs is due to upregulation of *dpp*, *gbb* or *dally* in ECs, we sequenced the mRNAs isolated from the purified GFP-labeled control and *piwiKD* ECs. Based on RNA sequencing and qRT-PCR results, *dpp* is significantly upregulated in the *piwiKD* ECs compared to the control ECs (Fig. 3H and 3I). Although RNA sequencing results show that *gbb* and *dally* are slightly upregulated in the *piwiKD* ECs (Fig. 3H), qRT-PCR results fail to confirm the finding (Fig. 3I). These results suggest that *dpp* upregulation in the *piwiKD* ECs might be responsible for germ cell differentiation defects.

To determine if *dpp* upregulation in the *piwiKD* ECs contributes to germ cell differentiation defects, we quantified SGCs outside the GSC niche in the germaria in which *piwi* and *dpp* are simultaneously knocked down in ECs. Here we used two different *piwi* (VDRC and HMS) and *dpp* RNAi (TRP and HMS) lines to knockdown *piwi* and *dpp* expression in ECs, respectively. Based on the numbers of the germaria carrying 3 or more SGCs, *c587*-driven *dpp* knockdown (TRP) can partially rescue the germ cell differentiation defects caused by *piwi* knockdown (VDRC) (Fig. 3J–L). *c587*-driven *piwi* knockdown by the HMS line yields stronger germ cell differentiation defects, which can be slightly and moderately repressed by *c587*-driven expression of TRP and HMS *dpp* RNAi lines, respectively (Fig. 3L and Fig. S4). *c587*-driven expression of the *dpp* HMS line causes partial GSC loss, but the expression of the *dpp* TRP line does not, suggesting that the HMS line might be stronger than the TRP line in knocking down *dpp* expression (Fig. S4). Based on the finding that the germ cell differentiation defects caused by *piwi* knockdown can only be partially repressed by *c587*-mediated *dpp* knockdown, the germ cell differentiation defects cannot be solely attributed to upregulated *dpp* expression in ECs (Fig. S4). Taken together, we propose that *dpp* upregulation in *piwiKD* ECs contributes to, but is not one of the major causing factors, for germ cell differentiation defects.

Defective EGFR-MAPK signaling in ECs causes germ cell differentiation defects by upregulating *dally* expression and thus increasing BMP signaling, and also prevents the formation of long cellular processes [17], [50]. Although our results show that Piwi knockdown does not lead to *dally* upregulation (Fig. 3H and 3I), we wanted to confirm if Piwi is required to maintain EGFR-MAPK signaling in ECs by examining the expression of pERK, a phosphorylated and active form of MAPK, in the *piwiKD* ECs. In the control, pERK is strongly and specifically expressed in all ECs, but not in cap cells and follicle cells (Fig. S5A). pERK is expressed at low levels in the remaining *piwiKD* ECs (Fig. S5A–E). Although pERK immunofluorescence intensity in the *piwiKD* ECs decreases by 25–65% in comparison with the control ECs (Fig. S5E), overall pERK levels might increase instead because the *piwiKD* ECs are often larger (Fig. S5A–D). To determine if increasing MAPK activity affects GSC maintenance and differentiation, we used *c587* to drive the expression of a kinase-active *rolled* (*rl*, encoding MAPK in *Drosophila*) mutant, *rl^SEM^*, in ECs [51]. Increasing MAPK activity does not have any obvious effect on GSC maintenance and CB differentiation (Fig. S5). Interestingly, following *c587*-mediated *piwi* knockdown by the three RNAi lines, ECs lose their long cellular processes (Fig. S6). These results align well with our earlier finding of no expression changes for *dally* in the *piwiKD* ECs, and also suggest that EGFR signaling is not the only pathway for maintaining EC cellular processes.

### Piwi is required in ECs for repressing TE activity and preventing DNA damage

**Figure 4:**
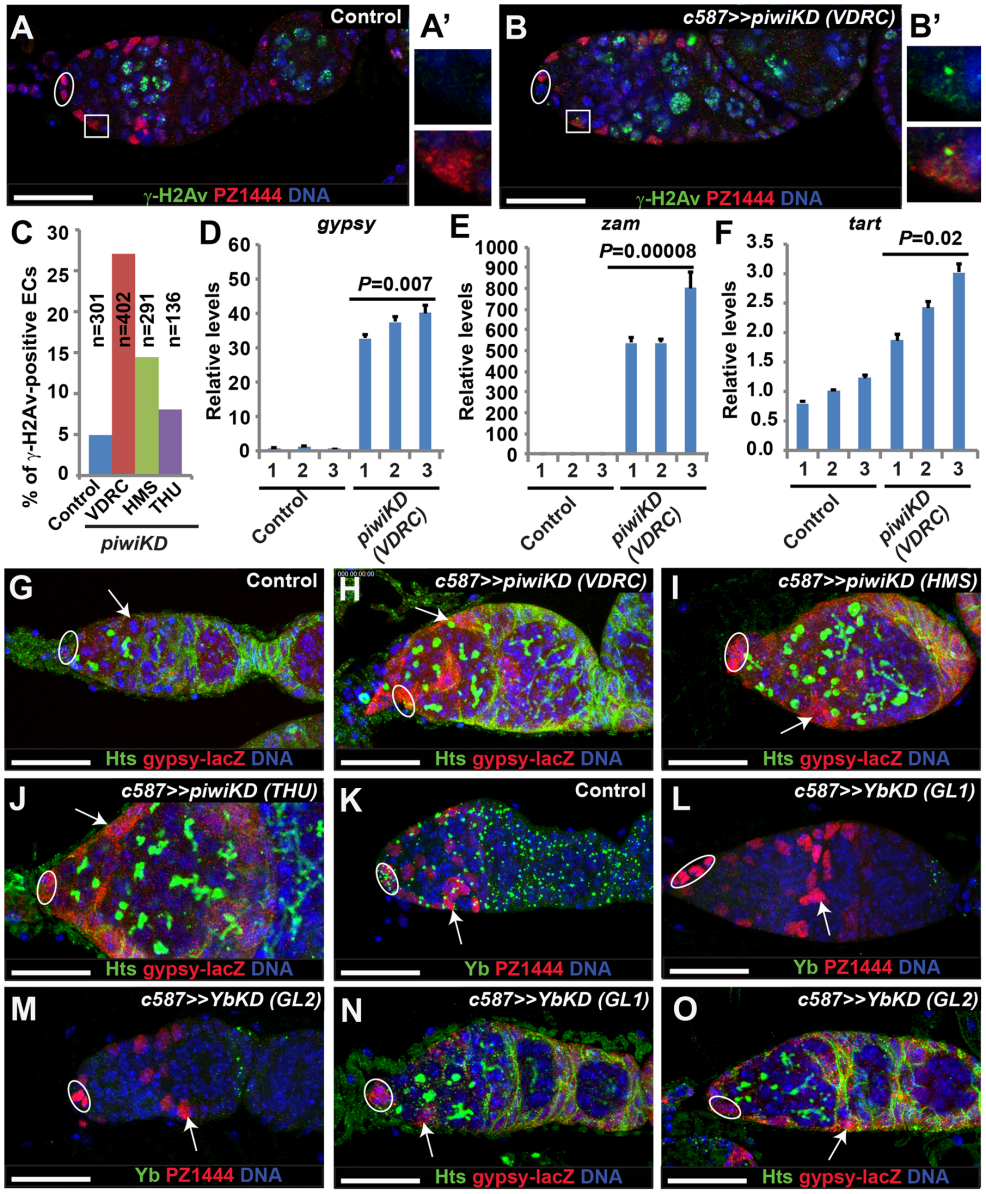
Piwi is required in ECs to repress transposon activity and thus prevent DNA damage. Ovals highlight cap cells. (**A**–**C**) Somatic *piwiKD* (**B**) causes an increase in γ-H2Av-positive and *PZ1444*-positive ECs in comparison with the control (**A**) in which *PZ1444*-positive ECs are negative for γ-H2Av. **A′** and **B′** highlight *PZ1444*-positive ECs in **A** and **B**, respectively. **C** represents quantitative results on γ-H2Av-positive ECs. (**D**–**F**) Quantitative RT-PCR results show that the transcripts for *gyspy* (**D**) and *zam* (**E**), but not *tart*, increase significantly in the *piwiKD* ECs in comparison with the control. (**G**–**J**) The *piwiKD* ECs (arrows, **H**–**J**) elevate *gypsy-lacZ* expression in comparison with the control ECs (arrow, **G**). (**K**–**M**) The *YbKD* ECs (arrows, **L** and **M**) lose Yb protein expression in comparison with the control ECs (arrow, **K**). (**N**, **O**) The *YbKD* ECs (arrows) elevate *gypsy-lacZ* expression. Scale bars: 25 µm.

piRNAs have been shown to be required for silencing TE activity in both germ cells and somatic cells [6], [7], [9], [52]. One of the outcomes for elevated TE activity is DNA damage. Thus, we examined the expression of phosphorylated H2Av (γ-H2Av), a *Drosophila* equivalent of mammalian H2AX [53], in the control and *piwiKD* ECs, and quantified γ-H2Av-positive ECs. γ-H2Av has been shown to be associated with DNA double-strand breaks in *Drosophila* cells [53]. In the control germaria, less than 5% of the ECs are positive for γ-H2Av (Fig. 4A and 4C). In contrast, 8%–25% of the *piwiKD* ECs are positive for γ-H2Av depending on the RNAi lines (Fig. 4B and 4C). These results indicate that Piwi is required in ECs to prevent DNA damage.

To further determine if Piwi is required in ECs for silencing TE activity, we sequenced the RNAs from the purified GFP-labeled control and *piwiKD* ECs by fluorescence-activated cell sorting (FACS). In this study, we chose to examine two common somatic cell-specific TEs, *gypsy* and *zam*, and a germline-specific TE, *tart* [54]. Both *gypsy* and *zam* transcripts are drastically and significantly upregulated in the *piwiKD* germaria in comparison with the control (Fig. 4D and 4E). As expected, the germline-specific *tart* transcripts are not changed dramatically in the *piwiKD* germaria in comparison with the control (Fig. 4F). In addition, we also used the *gypsy-lacZ* reporter to verify the qRT-PCR results. In the control germaria, *gypsy-lacZ* is not expressed (Fig. 4G). In contrast, it is dramatically upregulated in the *piwiKD* ECs by the three RNAi lines (Fig. 4H–J). These results further support the idea that Piwi is required in ECs to repress TE activity and prevent DNA damage.

Yb has been shown to regulate Piwi expression in TF and cap cells [27]. Indeed, in the *c587*-mediated *YbKD* germaria, Piwi protein expression in ECs and follicle cells is consistently downregulated (Fig. S7A–C′). However, Yb protein expression in somatic cells, including cap cells, ECs and early follicle cells, remains unchanged in the *c587*-mediated *piwiKD* germaria (Fig. S7D–G). To further determine if Yb is also required in ECs to repress TE activity, we examined the expression of *gypsy-lacZ* in the *YbKD* germarium. As previously reported, Yb is also expressed in all ovarian somatic cells, including ECs (Fig. 4K). c587-driven expression of two independent *Yb* RNAi lines can efficiently eliminate Yb expression in cap cells, ECs and early follicle cells (Fig. 4L and 4M). Interestingly, *gypsy-lacZ* expression is upregulated in the *YbKD* cap cells and ECs, indicating that Yb is also required in somatic cells to silence TEs (Fig. 4N and 4O). However, *gypsy-lacZ* expression appears to be lower in the *YbKD* ECs than in the *piwiKD* ECs (Fig. 4H–J, 4N and 4O). Although most of the *YbKD* germaria contain normal numbers of GSCs and SGCs (Fig. 4N and 4O), approximately 25% of the *YbKD* germaria carry 3 or more SGCs (Fig. S7H–K). These results suggest that Yb is also required in ECs to repress TEs and promote germ cell differentiation.

### Piwi is required intrinsically to maintain germ cells before adulthood

**Figure 5:**
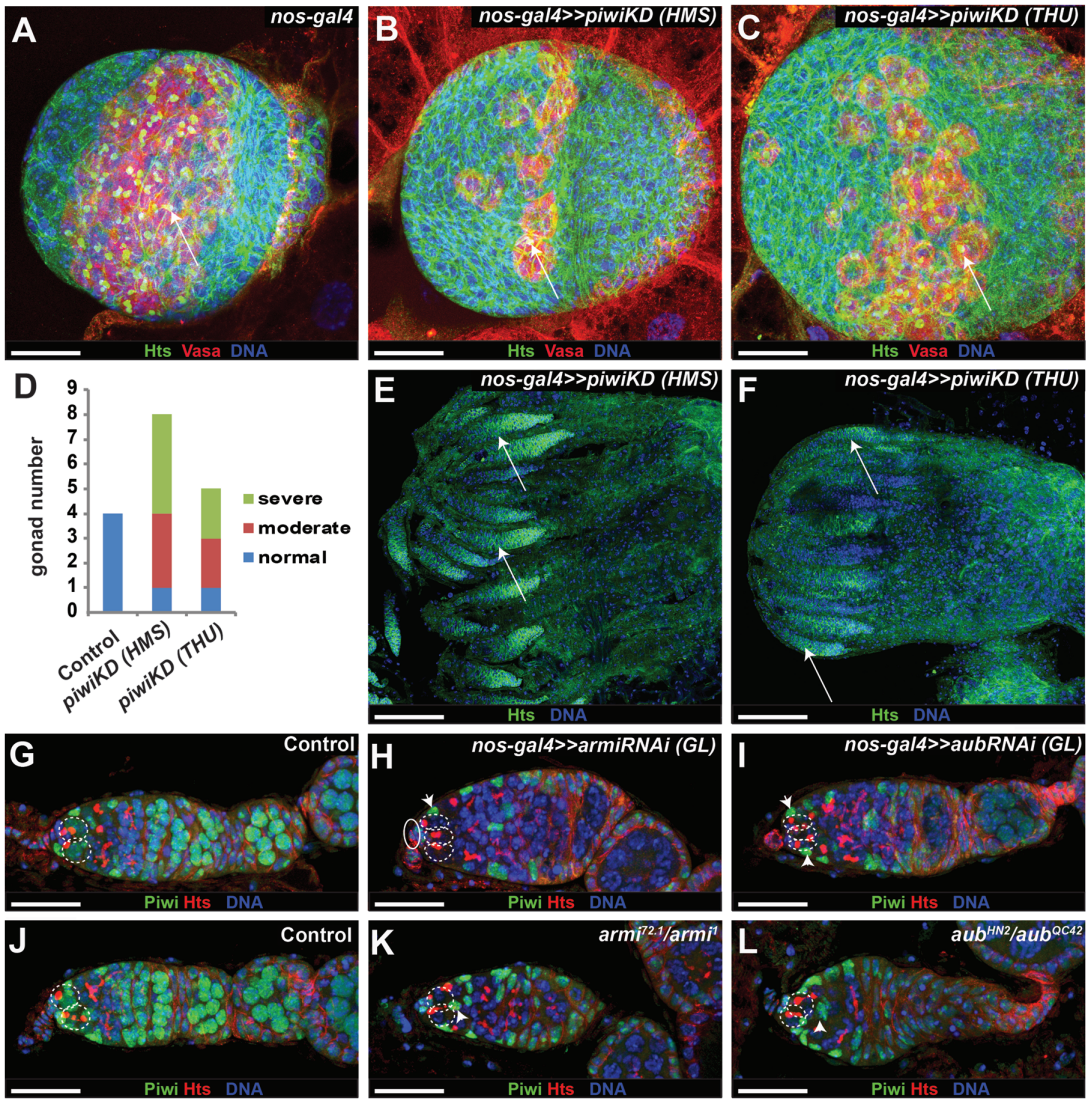
Piwi is required intrinsically to maintain PGCs or control GSC formation. (**A–D**) *nos-gal4*-driven *piwiKD* (**B**, **C**) leads to a reduction in PGC number in the third-instar larval gonads in comparison with the control (**A**). PGCs (arrows, **A**–**C**) are positive for Vasa (red) and also carry a spectrosome). **D** quantifies normal (**A**), moderate (**C**) and severe (**B**) phenotypes based on PGC numbers. (**E**, **F**) *nos-gal4*-driven *piwiKD* leads to complete germ cell loss in the germaria of the newly eclosed females, leaving empty germaria (arrows). (**G–I**) *nos-gal4*-driven *armi* (**H**) or *aub* (**I**) knockdown decreases nuclear Piwi expression in germ cells, but does not affect GSCs because the germaria still contain two or three GSCs (broken lines) as the control germarium (**G**). Nuclear Piwi expression remains in ECs (arrowheads) of the knockdown germaria (**H**, **I**). (**J–L**) *armi* (**K**) or *aub* (**L**) mutant germaria decrease nuclear Piwi expression in germ cells, but still have 2 or 3 GSCs as the control germarium (**J**). Nuclear Piwi expression remains in mutant ECs (arrowheads; **K**, **L**). Scale bars: 75 µm (**E** and **F**); 25 µm (**A–C** and **G–L**).

Since Piwi is expressed in all the germ cells, including GSCs, we then used *nanos-gal4VP16* (*nos-gal4*) to specifically knock down *piwi* in germ cells to determine if Piwi is also required intrinsically for GSC maintenance. The *nos-gal4* driver is expressed specifically in germ cells from primordial germ cells (PGCs) to adult germ cells, including GSCs [55]. In contrast with the control third-instar female gonad (Fig. 5A), *nos-gal4* driven expression of the two independent *piwi* RNAi strains, HMS and THU, leads to a reduction in PGC numbers in the female gonads, indicating that Piwi is required for PGC proliferation, maintenance or both (Fig. 5B–D). Furthermore, germ cell-specific *piwi* knockdown germaria in newly emerged adults show a complete loss of all germ cells, including GSCs (Fig. 5E and 5F). The GSC establishment takes place at the transitional period from the third instar-larval stage to the pupal stage. These results indicate that Piwi is required intrinsically to control PGC maintenance and/or GSC establishment.

To further explore whether other piRNA components are also required for GSC maintenance before adulthood, we used *nos-gal4* driven expression of RNAi against *armi* and *aub* to inactivate their function throughout germ cell development. Germ cell-specific *armi* or *aub* knockdown by two independent RNAi lines for each gene leads to a dramatic reduction in nuclear Piwi protein expression in germ cells, but does not affect nuclear Piwi expression in somatic cells (Fig. 5G–I). In addition, germline-specific *armi* or *aub* knockdown also causes the full penetrance of female sterility. These results suggest that both of them are efficiently knocked down in the germline because they are required for Piwi nuclear localization and to prevent the activation of meiotic cell cycle checkpoints caused by transposon-induced DNA damage [56]. However, the *armi* or *aub* knockdown germaria from newly eclosed females still contain two or three GSCs in their germaria, indicating that they are not required intrinsically for early germ cell development and GSC formation (Fig. 5G–I). Consistently, newly eclosed *armi^72.1^/armi^1^* and *aub^HN2^/aub^QC42^* mutant females also maintain two or three GSCs in their germaria, and dramatically decrease nuclear Piwi expression in GSCs and their progeny (Fig. 5J–L). Since these mutants carry strong loss-of-function mutations in *armi* or *aub* [56], [57], these results further support that Armi and Aub are dispensable for germ cell development before adulthood. Taken together, our results indicate that Piwi controls early germ cell development, GSC formation or both independently of Armi and Aub.

### Piwi is required intrinsically to control GSC maintenance and germ cell differentiation in the adult ovary

**Figure 6:**
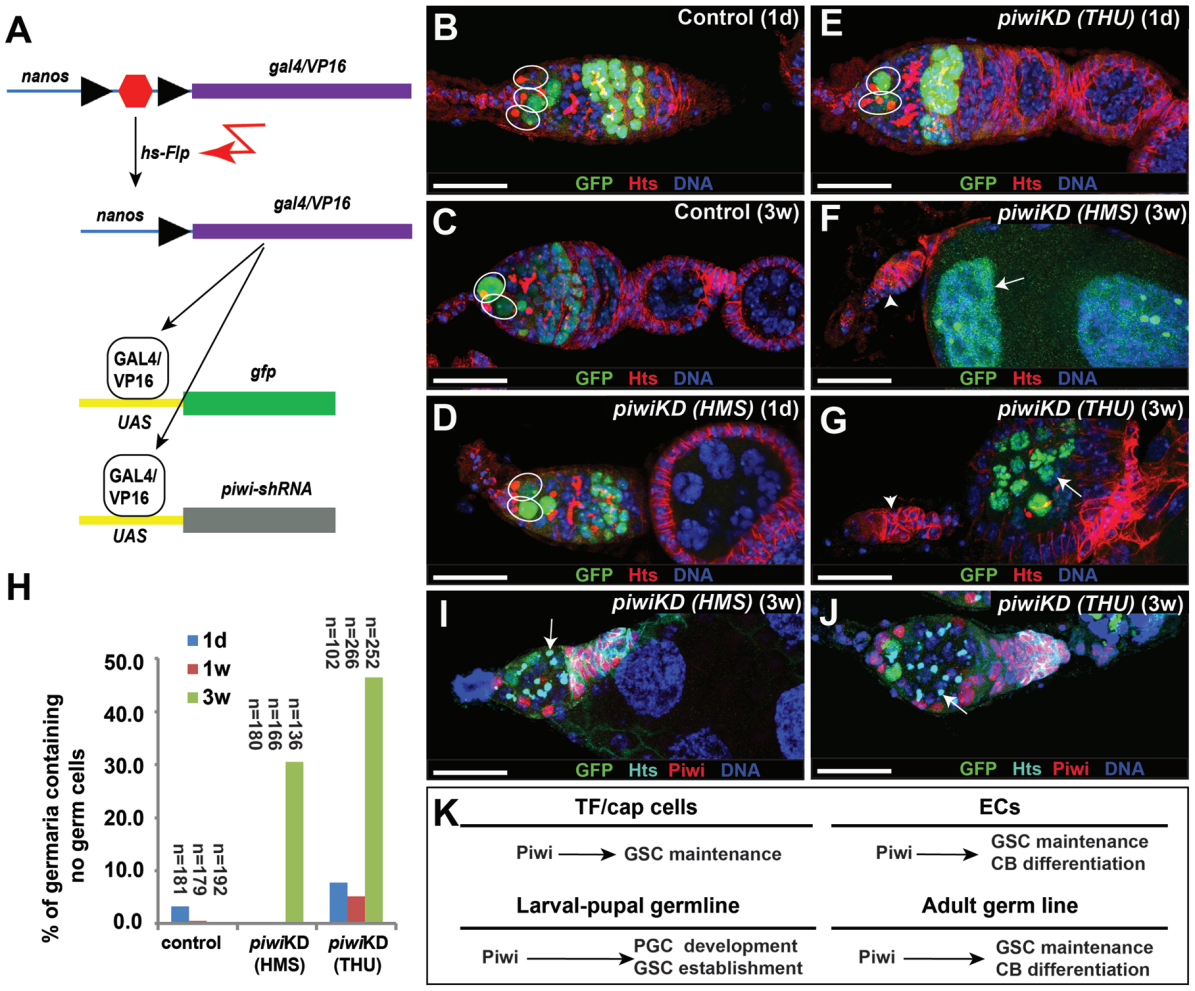
Piwi is required intrinsically to maintain GSCs and promote germ cell differentiation. (**A**) A flip-out strategy for *nos-gal4*-driven *piwi* knockdown specifically in adult GSCs and their progeny, which are also labeled by GFP expression. (**B**, **C**) GFP-marked control GSCs (circles) detected 1 d AHS (**B**) are still maintained 3 w AHS (**C**). (**D–H**) GFP-marked *piwiKD* GSCs (circles) detected 1 d AHS (**D**, **E**) are lost 3 w AHS (**F**, **G**). Consequently, the *piwiKD* germaria (arrowheads) completely lose their germ cells, and some marked GSCs have developed into GFP-positive egg chambers (arrows). **H** represents the quantitative results on the germaria containing no germ cells. (**I**, **J**) *piwiKD* germaria accumulate excess SGCs (arrow), which are negative for Piwi protein though GFP-negative, outside the GSC niche 3w AHS. As expected, all somatic cells are still positive for Piwi. (**K**) A working model for the roles of Piwi in TF/cap cells, ECs and germ cells. Scale bars: 25 µm.

To determine if Piwi is required in the adult germline to maintain GSCs, we used the flip-out system, in which a transcriptional stop sequence flanked by two FRT sites is inserted between the *nanos* promoter and *gal4VP16*, to activate the expression of RNAi lines along with the GFP reporter specifically in germ cells after heatshock treatments of adult females (Fig. 6A). In the control ovaries, GFP-positive GSCs detected 1 day after heatshock (1 d AHS) remain in the niche for additional three weeks (Fig. 6B and 6C). The GFP-marked *piwiKD* GSCs can be readily detected in the germaria 1 d AHS (Fig. 6D and 6E). In contrast, most of the GFP-marked *piwiKD* GSCs are lost three weeks AHS, and consequently over 30% of the *piwiKD* germaria have completely lost GSCs (Fig. 6F–H). In addition, more undifferentiated SGCs also accumulate in the *piwiKD* germaria three weeks AHS, indicative of germ cell differentiation defects (Fig. 6I and 6J). Interestingly, some SGCs outside the GSC niche are GFP-negative and also Piwi-negative, which is caused by the failure in *nos-gal4*-driven GFP expression due to an unknown reason (Fig. 6I and 6J). These results demonstrate that Piwi is required in adult germline for GSC maintenance and germ cell differentiation.

## Discussion

Although the primary piRNA pathway is known to operate in *Drosophila* ovarian somatic cells to repress TE activity, its biological importance in *Drosophila* oogenesis is not well understood. Piwi, one of the key components in the primary piRNA pathway, has been shown to function in TF/cap cells to control GSC maintenance [22], [23], [24]. In this study, we have revealed a novel role of Piwi in ECs to control GSC lineage differentiation and additional roles in the germline for PGC and GSC maintenance (Fig. 6K). *piwi* knockdown in somatic cells results in defective GSC maintenance, defective germ cell differentiation as well as increased TE activity and DNA damage. *dpp* upregulation contributes to the germ cell differentiation defects caused by somatic *piwi* knockdown. A recent study has also come to a similar conclusion that Piwi is required in somatic cells for promoting germ cell differentiation by repressing BMP signaling [58]. In addition, we have also shown that Piwi is required in PGCs to control PGC maintenance, GSC formation or both, and is also required in adult germline to maintain GSCs and promote germ cell differentiation (Fig. 6K). Therefore, our genetic results argue strongly that Piwi functions in germline to maintain PGCs and GSCs as well as to promote germ cell differentiation. Therefore, we have revealed new functions of Piwi in multiple cell types to maintain GSCs and promote germ cell differentiation (Fig. 6K).

### Piwi is required in ECs and germ cells to promote germ cell differentiation

Recent studies have shown that ECs play an important role in promoting germ cell differentiation by repressing BMP signaling [16]. Thus far, genes identified to be important in ECs for germ cell differentiation repress the expression of either *dally* or *dpp*, thereby preventing BMP signaling in ECs. EGFR signaling has been proposed to be responsible for directly repressing *dally* expression in ECs, but is dispensable for EC survival [17]. In addition, recent studies have also shown that Rho signaling and Eggless are also required in ECs for repression of *dally* expression, and are also required for EC survival and the maintenance of long EC cellular processes [18], [19]. Lsd1, Rho signaling and Eggless have been shown to be required to repress *dpp* transcription [18], [19], [20]. In this study, we have also shown that Piwi is required in ECs for *dpp* repression but is dispensable for *dally* repression. In addition, it is also required in ECs for maintaining their survival and long cellular processes. Our genetic results suggest that *dpp* upregulation partially contributes to the germ cell differentiation defects caused by Piwi knockdown in ECs. In contrast with the recent study claiming that *dpp* upregulation is responsible for the germ cell differentiation defects caused by defective Piwi in ECs [58], our study shows that *dpp* upregulation is not the main cause for the germ cell differentiation defects caused by defective ECs. In our study, we used two independent RNAi lines against *piwi* and *dpp*, respectively, to show that *dpp* knockdown does not drastically rescue the germ cell differentiation defects caused by *piwi* knockdown ECs. Furthermore, the *dpp* levels remain low in *piwi* knockdown ECs even after upregulation based on RNA sequencing results. Thus, we conclude that *dpp* upregulation in *piwi* knockdown ECs is only partially responsible for the germ cell differentiation defects. In addition, Piwi is required in somatic ovarian cells to repress TE activity and prevent transposon-induced DNA damage. However, it remains unclear if *dpp* upregulation and the loss of ECs and their long cellular processes are caused by DNA damage, and how Piwi is involved in repressing BMP signaling activity in differentiated germ cells via repression of *dpp* expression in ECs.

Piwi has previously been demonstrated to be required intrinsically for promoting GSC division [27]. Piwi is expressed in GSCs and their differentiated progeny [27]. This study has shown that germline-specific knockdown of Piwi function in the adult ovary leads to the accumulation of undifferentiated single germ cells, revealing a new intrinsic role of Piwi in controlling germ cell differentiation (Fig. 6K). Piwi has been shown to be involved in the piRNA pathway and epigenetic regulation. In the future, it will be important to determine if the piRNA pathway, epigenetics or both play a role in the regulation of germ cell differentiation.

### Piwi is required in both somatic cells and germ cells to maintain GSC lineage

Although Piwi is generally expressed in almost all somatic cells and germ cells of the *Drosophila* ovary, the previous studies proposed that Piwi acts in TF/cap cells to control GSC self-renewal [22], [23], [24]. In this study, we have confirmed the somatic role of Piwi in GSC maintenance, and have also revealed new roles of Piwi to maintain PGCs before adulthood and GSCs after adulthood. In addition, our temperature shift experiments have shown that Piwi is also required in adult somatic cells, TF/cap cells, ECs or both, to maintain GSCs. Interestingly, RNAi-mediated knockdown of Piwi function in adult somatic cells only produces a moderate GSC loss phenotype in comparison with the severe GSC loss phenotype of *piwi* mutants, suggesting that Piwi might also function in other cell types to maintain GSCs. In the future, it will be important to determine how Piwi functions in TF/cap cells and ECs to maintain GSC self-renewal. Therefore, our study has not only confirmed the somatic role of Piwi in controlling GSC self-renewal but also has suggested its function in additional cell types to maintain GSCs (Fig. 6K).

Although Piwi has been shown to be required to control PGC formation and GSC division [25], [27], it remains unclear if Piwi is required intrinsically to maintain PGCs and GSCs. In this study, we have revealed critical roles of Piwi in different developmental stages of germ cells. First, Piwi is required in the developing female gonad to control PGC proliferation, survival or both because germline-specific Piwi knockdown leads to a reduction in PGC number in third-instar female larval gonads. Second, Piwi is required in PGCs to control their survival or GSC formation because germline-specific *piwi* knockdown leads to a complete elimination of germ cells including GSCs in newly eclosed adult females. Interestingly, germline-specific knockdown of either *armi* or *aub*, two of which work with *piwi* to control piRNA biogenesis, fails to produce any GSC loss phenotype in newly eclosed adult females, suggesting that Piwi controls PGC proliferation and survival or GSC formation possibly independently of Armi and Aub, possibly piRNAs. Piwi has been shown to physically interact with HP1a to epigenetically control gene expression in somatic tissues [41]. In addition, *piwi* genetically interacts with *certo*, encoding a chromodomain-containing protein, to control GSC maintenance [59]. Our findings are consistent with the notion that Piwi controls early germ cell development perhaps via epigenetics. Third, Piwi is required in adult GSCs for their maintenance because germline-specific knockdown in the adult ovary also causes a moderate GSC loss phenotype. Therefore, we propose that Piwi functions in multiple stages of germline development to control PGC proliferation and survival, and GSC maintenance (Fig. 6K).

## Materials and Methods

### Drosophila strains and culture

The *Drosophila* stocks used in this study include: *c587* [35], *PZ1444* [37], *UAS-dcr2*, *UAS-dppRNAi* lines (TR00047P.1;HMS00011), *UAS-piwiRNAi* lines (VDRC101658, HMS00606 and THU00412), *UAS-armiRNAi* lines (GL00254; HMS00098), *UAS-aubRNAi* lines (GL00076; HMS00611) and *UAS-YbRNAi* (GL00053; GL00204). *Drosophila* strains were maintained and crossed at room temperature on standard cornmeal/molasses/agar media unless specified. To maximize the RNAi-mediated knockdown effect, newly eclosed flies were cultured at 29°C for a week before the analysis of ovarian phenotypes.

### Construction of UAS-RNAi and nos>mCherry SV40 polyA>gal4VP16 Strains

The new *THU UAS-RNAi* line targeting *piwi* was constructed using the pVALIUM20 vector according to the published procedure [60]. The targeting sequence for *piwi* is CCCGGTCATGCTGCAGACGAA, which was designed based on the algorithm of DSIR.

To construct *pnos-FRT-mCherry-SV40 polyA-FRT-gal4VP16-nos 3′UTR*, different components were assembled together by five steps. First, the coding region of *mCherry* was amplified from a *mCherry*-containing vector using 5′-acgctagctatggtgagcaagggcgaggag-3′and 5′-gactcgagttacttgtacagctcgtccat-3′ primers (Nhe I and XhoI sites underlined), and was cloned into NheI-XhoI sites of the *pFRT-SV40 polyA-FRT* vector (a gift from Elizabeth R. Gavis). Then, the *FRT-mCherry* fragment amplified using 5′-atcatatgggggatcttgaagttcctatt-3′ and 5′-gactcgagttacttgtacagctcgtccat-3′ primers (Nde I and XhoI sites underlined) from the *pFRT-mCheery-SV40 polyA-FRT* was cloned into the pGEM-T vector (Promega) to generate *pFRT-mCherry*. Second, the *SV40 polyA-FRT* fragment amplified from the *pFRT-SV40 polyA-FRT* vector using 5′-gactcgagggtacctctagaggatctttgtga-3′ and 5′-atgcggccgccatatgcaaaagcgctctgaagttcctatact-3′ primers (XhoI and NotI NdeI sites underlined) was cloned into the XhoI-NotI sites of *pFRT-mCherry* to generate *pFRT-mCheery-SV40 polyA-FRT*. Third, the *EGFP* coding region amplified from *pEGFP-N3* (Clontech) using 5′-tcgaattccatcgccaccatggtgagcaa-3′ and 5′-tacagatctcttgtacagctcgtccatgccga-3′ primers (EcoR I and BglII sites underlined) was cloned into the BglII-EcoRI sites of *pUAST-attB* [61] to generate *pEGFP-attB*. Fourth, the NotI flanked 3.13 Kb fragment from *pCSpnosFGVP* (a gift from Elizabeth R. Gavis) containing *nos promoter-ATG (NdeI-start codon) gal4VP16-nos 3′UTR* was subcloned into two NheI sites of *pEGFP-attB* to generate *pnos-NdeI-gal4VP16-nos 3′UTR-attB*. Finally, the NdeI flanked *pFRT-mCherry-SV40 polyA -FRT* fragment from *pFRT-mCheery-SV40 polyA-FRT* was subcloned into the NdeI site of *pnos-NdeI-gal4VP16-nos 3′UTR-attB* to generate *pnos-FRT-mCherry-SV40 polyA-FRT-gal4VP16-nos 3′UTR*, which was introduced into an *attP* site-containing fly strain (BL#24482) using PhiC31 integrase-mediated transgenesis by BestGene, Inc.

### Immunohistochemistry

Immunohistochemistry was performed according to our previously published procedures [62], [63]. The following antibodies were used in this study: rabbit polyclonal anti-β-galactosidase antibody (1∶100, Cappel), Guinea pig polyclonal anti-Piwi antibodies (1∶100; produced by H. Lin), chicken polyclonal anti-GFP antibody (1∶200, Jax), mouse monoclonal anti-Hts antibody (1∶50, DSHB), mouse monoclonal anti-Yb antibody (1∶200; kindly provided by Dr. H. Siomi), rabbit polyclonal anti-pS137 H2Av antibody (1∶100, Rockland), rabbit monoclonal anti-pS423/425 Smad3 antibody (1∶100, Epitomics), rabbit polyclonal anti-pERK antibodies (1∶25, Cell Signaling) and rat monoclonal anti-Vasa antibody (1∶50, DSHB). All images were taken with a Leica TCS SP5 confocal microscope.

### Cell sorting and RNA sequencing

*Drosophila* ovaries were dissected and placed in Grace's medium (Sigma-Aldrich; G9771) and then washed twice by adding 1× DPBS and centrifuged at 700×g for 1 minute. The ovaries were incubated with prewarmed Collagenase (Worthington, Collagenase Type II, Lot# 50D11833) in 15 ml conical tube at 37°C water bath for 3 minutes with gentle shaking. Enzyme reaction was stopped after 3 minutes by adding cold 1× DPBS+2% FBS. Dissociated sample was washed by adding 1× DPBS and centrifuged at 700×g, 4°C for 5 minutes. The cell pellet was resuspended in 1× DPBS and filtered with 70 um Filcon (BD; 340605) in to 5 ml flow tubes. Cells were centrifuged and then resuspended in 200 ul of 1× DPBS for sorting at 45 psi with 70 um tip (BD; InFlux) immediately in to TRIzol (life technologies; 15596-018). Total RNAs were extracted with Trizol and purified by organic extraction followed by isopropanol precipitation.

Following manufacturer's directions and using the Illumina TruSeq library construction kits (Illumina, Cat. No. RS-122-2001/2), mRNA was isolated from 150 ng of total RNA per sample and short fragment libraries were constructed. The resulting libraries were purified using Agencourt AMPure XP system (Backman Coulter, Cat. No. A63880), and were then quantified using a Bioanalyzer (Agilent Technologies) and a Qubit Fluorometer (Life Technologies). All libraries were pooled, re-quantified and run as 50 bp single-end lanes on an Illumina HiSeq 2000 instrument, using HiSeq Control Software 1.5.15.1 and Real-Time Analysis (RTA) version 1.13.48.0. Secondary Analysis version CASAVA-1.8.2 was run to demultiplex reads and generate FASTQ files.

For qRT-PCR, total RNAs were first treated by DNase I, and were then used for synthesis of cDNAs using mixed oligo dT and random primers and SuperScript III Reverse Transcriptase (Life Technologies). Fluorescence-based quantitative real-time PCR (qPCR) was performed to quantify *gypsy*, *zam*, *TART*, *gbb*, *dpp* and *dally* with *tbp*, *gapdh* and *rpl32* as internal controls using primers shown in Table S1. After cDNAs were diluted at 1∶100, 2 µl aliquots of each cDNA sample were added to 5 ul of 2× power SYBR Green PCR Master Mix (Applied Biosysterms part No.: 4367659, Lot No. :1305403), 0.5 µl each of 10 nm Forward & Reverse primer and 2 ul of water in a 384-well plate. The resulting reactions were run on an ABI 7900HT according to the instruments standard protocol. Analysis of the fluorescence curves was done using ABI's SDS2.4 software. The Ct values were analyzed using the Biogazelle qBase Plus version 2.4 software to generate normalized relative quantities using assays for endogenous controls.

## Supporting Information

Figure S1 **Piwi knockdown increases apoptosis in ECs.** (**A**) PZ1444-positive control ECs are negative for TUNEL labeling. (**B**, **C**) Apoptotic PZ1444-positive ECs (arrows) are detected in the piwiKD germaria by VDRC (**B**) and HMS (**C**) RNAi lines. The dying ECs appear to show low PZ1444 expression. (**D**) Quantification results of TUNEL-positive ECs in control and *piwiKD* germaria. Scale bars: 25 µm.
(TIF)

Figure S2 ***c587*** **drives expression of** ***piwi*** **RNAi in adult cap cells.** (**A, A′**) Piwi is expressed in cap cells (broken lines) at low levels. (**B**–**D′**) *c587*-driven expression of VDRC (**B, B′**), HMS (**C, C′**) and THU (**D, D′**) *piwi* RNAi lines reduces Piwi protein expression in adult cap cells as well as in ECs. Scale bars: 25 µm.
(JPG)

Figure S3 **Piwi is required in ECs to prevent BMP signaling in differentiated germ cells.** Cap cells are highlighted by asterisks. (**A**, **B**) *c587*-mediated *piwiKD* by the THU line results in upregulated pMad (**A**) and Dad-lacZ (**B**) expression in SGCs a few cells away from cap cells. (**C**) *bam-GFP* is repressed in GSCs and upregulated in differentiated germ cell cysts (arrow) of the control germarium. (**D**–**F**) *c587*-mediated *piwiKD* by three *piwi* RNAi lines causes repression of *bam-GFP* expression in some SGCs (arrowheads) outside the GSC niche. Differentiated cysts (arrows) still maintain high *bam-GFP* expression. **G** shows quantification results of *bam-GFP*-negative CBs. Scale bars: 25 µm.
(JPG)

Figure S4 ***dpp*** **upregulation in** ***piwiKD*** **ECs might not be the major factor causing germ cell differentiation defects.** Asterisks indicate the GSC niche. (**A–C**) *c587*-mediated *dpp* knockdown by TRP (**A**) and HMS (**B**, **C**) lines does not affect GSC maintenance and differentiation because the knockdown germaria still maintain two GSCs (arrows). However, some *dppKD* germaria (**C**) by the HMS line, but not by the TRP line, completely lose their germ cells including GSCs. (**D–H**) *c587*-mediated *dpp* knockdown suppresses the germ cell differentiation defects in some *piwiKD* germaria (**E**, **G**) but not in the other germaria (**F**, **H**) in comparison with the germ cell differentiation defects in the *piwiKD* germaria (**D**). Arrows in **D**, **F** and **H** point to spectrosomes, whereas those in **E** and **G** indicate branched fusomes. Scale bars: 25 µm.
(TIF)

Figure S5 **pERK activity in** ***piwiKD*** **ECs.** (**A**) pERK is specifically expressed in ECs (one by arrowhead) of the control germarium. (**B**–**E**) *c587*-mediated *piwiKD* ECs (arrowheads) are often larger and show lower pERK fluorescence intensity. **E** shows quantification results on pERK intensity. (**F**–**G**) *c587*-mediated *rl^SEM^* expression does not affect GSC and CB numbers (arrows indicate GSCs). **H** shows that there are no significant differences in GSCs and CBs between control and *rl^SEM^* -expressing germaria. Scale bars: 25 µm.
(TIF)

Figure S6 **Piwi knockdown in ECs disrupts the formation of their long cellular processes.** (**A**) *c587*-mediated *CD8GFP* expression highlights long EC cellular processes (arrows) wrapping CBs, mitotic cysts and 16-cell cysts in the control germarium. (**B–D**) In the *c587*-mediated *piwiKD* germaria by three RNAi lines, *HMS* (**B**), *THU* (**C**) and *VDRC* (**D**), there are no long-GFP-positive cellular processes wrapping differentiated germ cells. Scale bars: 25 µm.
(TIF)

Figure S7 **Yb is required in ECs to promote germ cell differentiation.** The GSC niche is highlighted by broken lines (**A**–**C′**) or the asterisk (**H**–**J**). (**A**–**C′**) *c587*-mediated *YbKD* by two RNAi lines, *GL1* (**B**, **B′**) and *GL2* (**C**, **C′**), leads to a Piwi protein expression reduction in cap cells (broken lines), ECs (arrowheads) and early follicle cells in comparison with the control (**A**, **A′**). (**D–G**) *c587*-mediated *piwiKD* by three RNAi lines, *VDRC* (**E**), *HMS* (**F**) and *THU* (**G**), has no effect on YB protein expression in cap cells, ECs and early follicle cells in comparison with the control (**D**). (**H**) The control germarium contains three GSCs and differentiated cysts (arrow). (**I–K**) *c587*-mediated *YbKD* causes an accumulation of excess SGCs (arrowheads) in the germarium. **K** represents the quantitative results on the germaria carrying three or more SGCs. Scale bars: 25 µm.
(TIF)

Table S1 **This table contains the nucleotide sequences of all the primers used in this study.**
(DOCX)
