# Supplementary figures and images for: Piwi Is Required in Multiple Cell Types to Control Germline Stem Cell Lineage Development in the Drosophila Ovary
Source: PLoS One. 2014 Mar 21;9(3):e90267. doi: 10.1371/journal.pone.0090267 (PMC3962343; doi:10.1371/journal.pone.0090267)

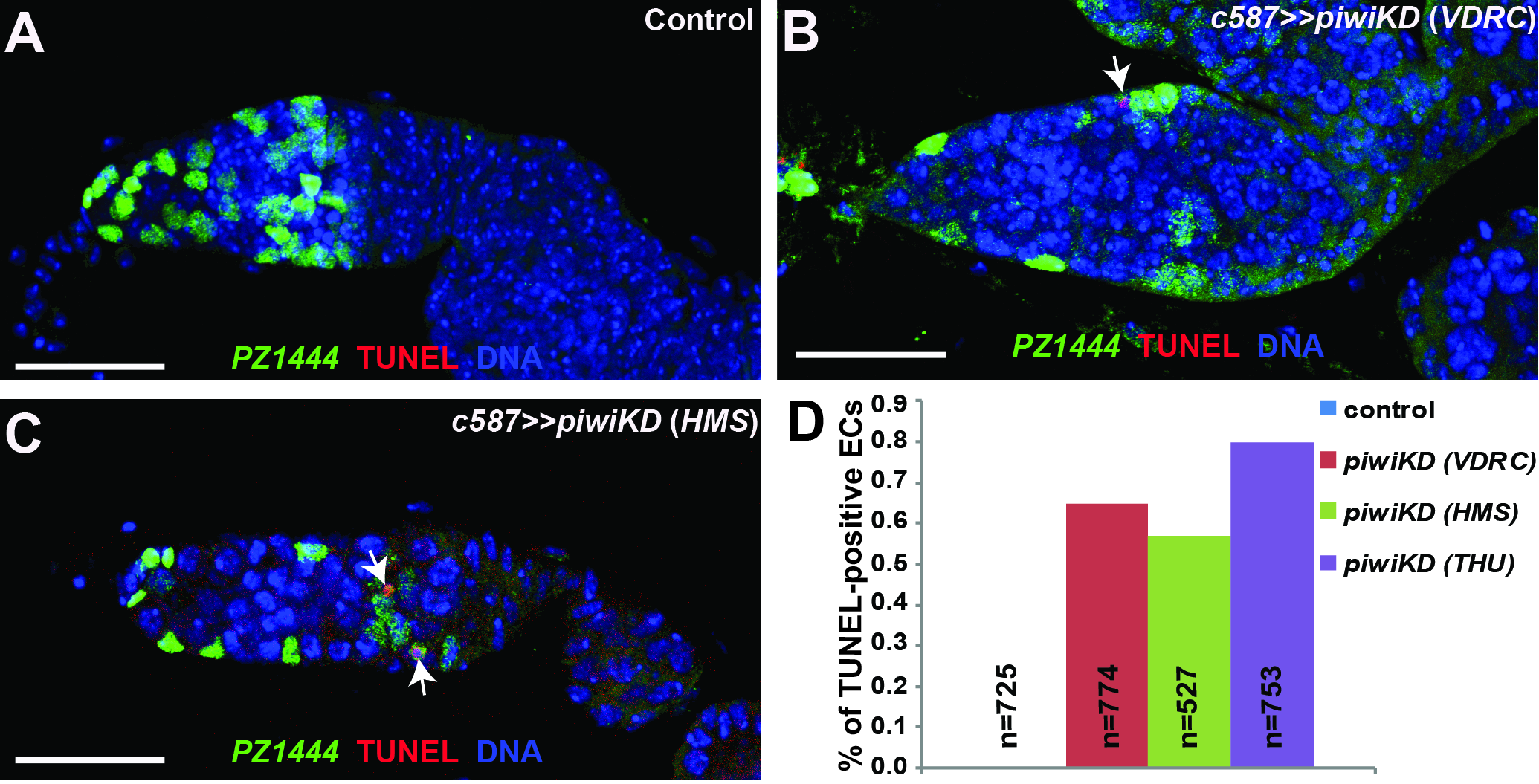

Supplement: Figure S1 — Piwi knockdown increases apoptosis in ECs. (A) PZ1444-positive control ECs are negative for TUNEL labeling. (B, C) Apoptotic PZ1444-positive ECs (arrows) are detected in the piwiKD germaria by VDRC (B) and HMS (C) RNAi lines. The dying ECs appear to show low PZ1444 expression. (D) Quantification results of TUNEL-positive ECs in control and piwiKD germaria. Scale bars: 25 µm. (TIF) [file pone.0090267.s001.tif]

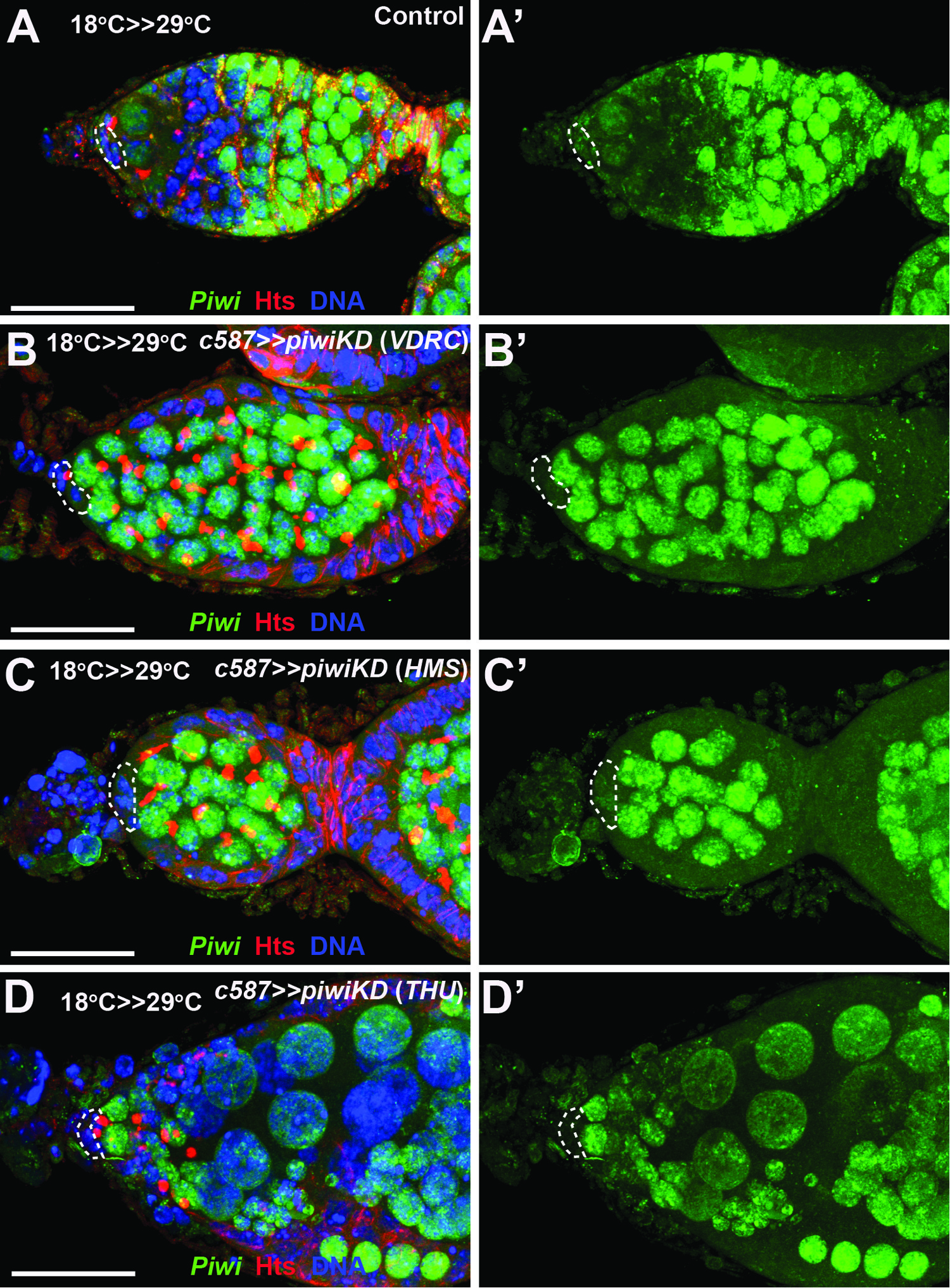

Supplement: Figure S2 — c587 drives expression of piwi RNAi in adult cap cells. (A, A′) Piwi is expressed in cap cells (broken lines) at low levels. (B–D′) c587-driven expression of VDRC (B, B′), HMS (C, C′) and THU (D, D′) piwi RNAi lines reduces Piwi protein expression in adult cap cells as well as in ECs. Scale bars: 25 µm. (JPG) [file pone.0090267.s002.jpg]

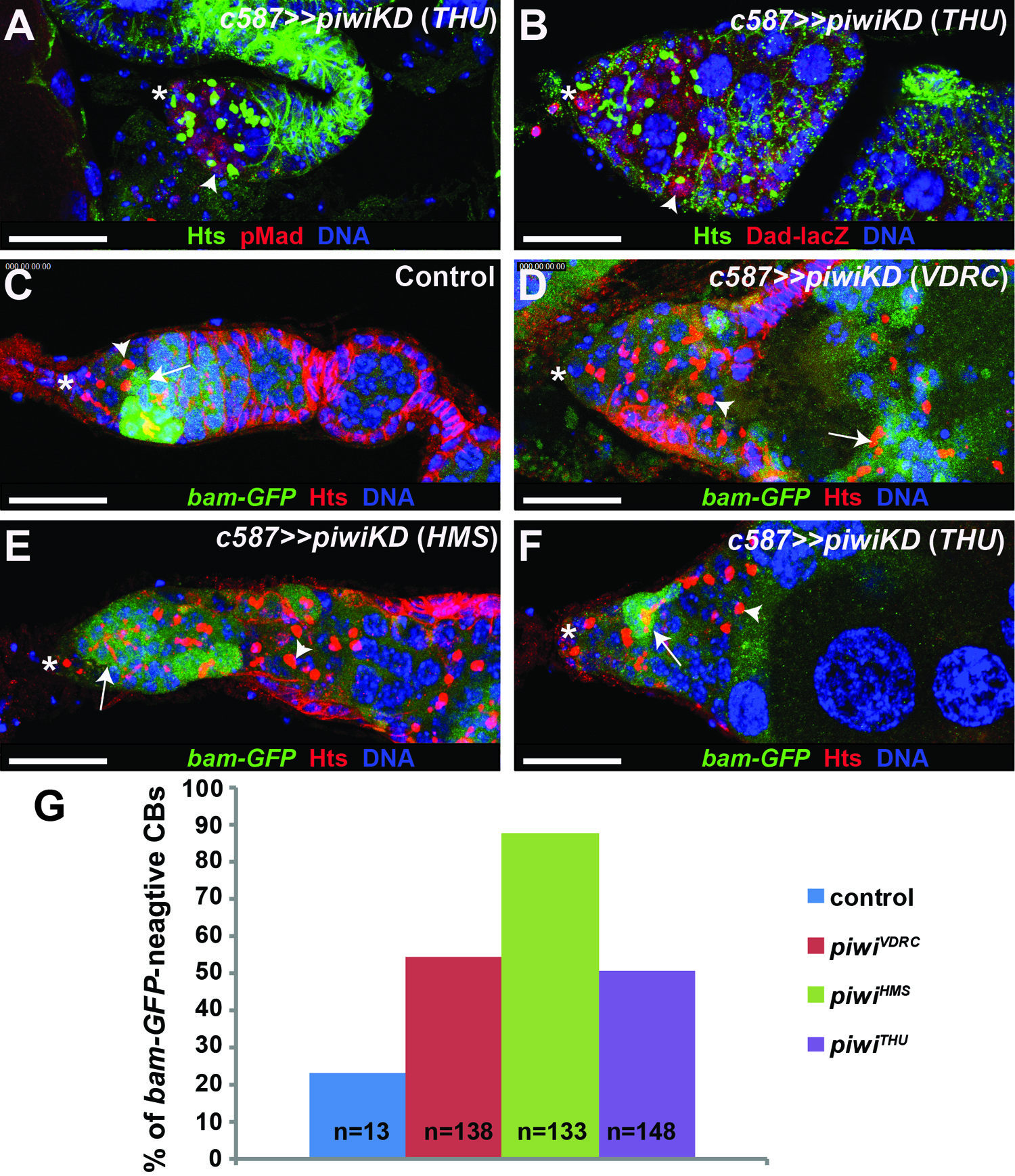

Supplement: Figure S3 — Piwi is required in ECs to prevent BMP signaling in differentiated germ cells. Cap cells are highlighted by asterisks. (A, B) c587-mediated piwiKD by the THU line results in upregulated pMad (A) and Dad-lacZ (B) expression in SGCs a few cells away from cap cells. (C) bam-GFP is repressed in GSCs and upregulated in differentiated germ cell cysts (arrow) of the control germarium. (D–F) c587-mediated piwiKD by three piwi RNAi lines causes repression of bam-GFP expression in some SGCs (arrowheads) outside the GSC niche. Differentiated cysts (arrows) still maintain high bam-GFP expression. G shows quantification results of bam-GFP-negative CBs. Scale bars: 25 µm. (JPG) [file pone.0090267.s003.jpg]

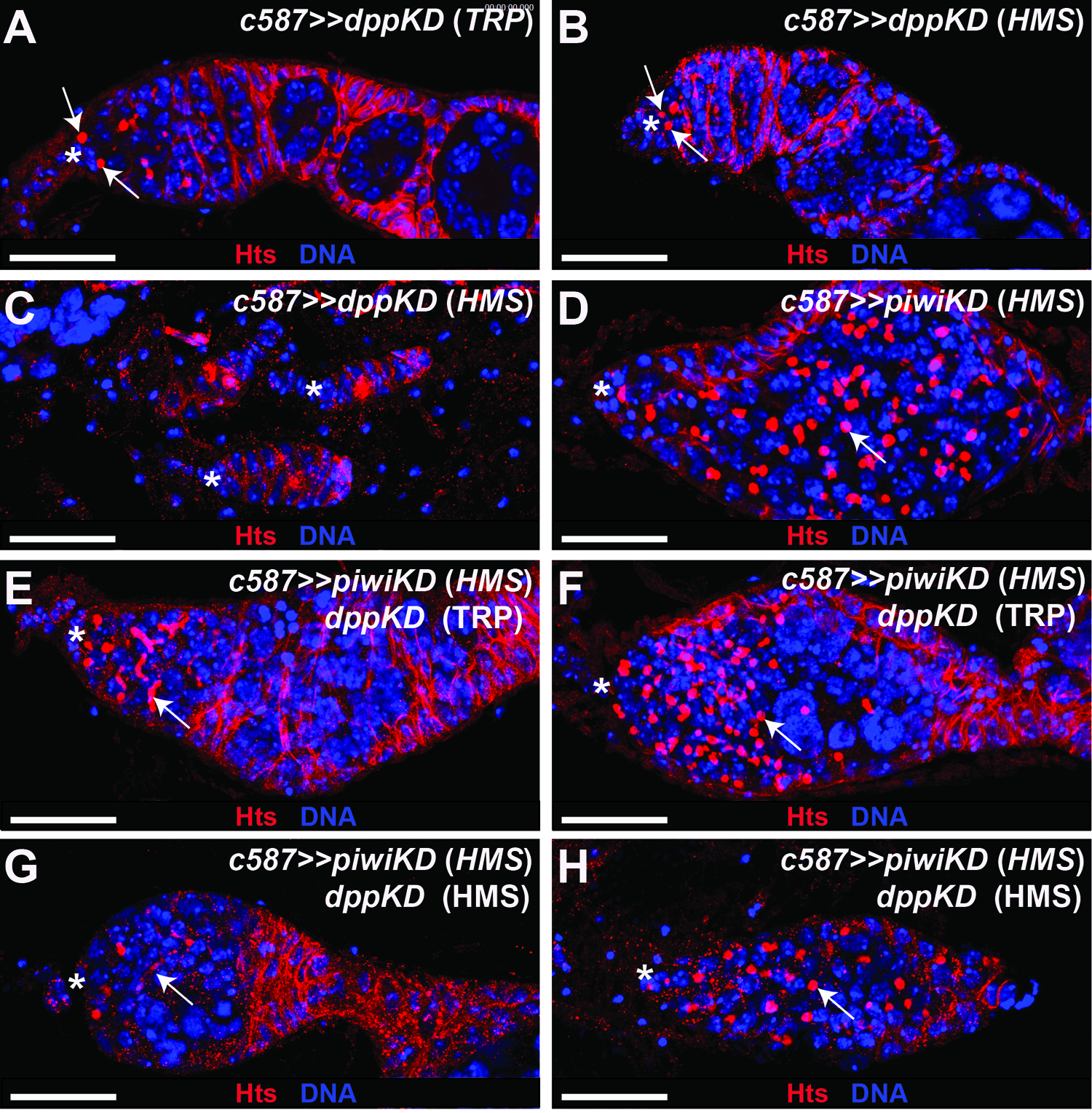

Supplement: Figure S4 — dpp upregulation in piwiKD ECs might not be the major factor causing germ cell differentiation defects. Asterisks indicate the GSC niche. (A–C) c587-mediated dpp knockdown by TRP (A) and HMS (B, C) lines does not affect GSC maintenance and differentiation because the knockdown germaria still maintain two GSCs (arrows). However, some dppKD germaria (C) by the HMS line, but not by the TRP line, completely lose their germ cells including GSCs. (D–H) c587-mediated dpp knockdown suppresses the germ cell differentiation defects in some piwiKD germaria (E, G) but not in the other germaria (F, H) in comparison with the germ cell differentiation defects in the piwiKD germaria (D). Arrows in D, F and H point to spectrosomes, whereas those in E and G indicate branched fusomes. Scale bars: 25 µm. (TIF) [file pone.0090267.s004.tif]

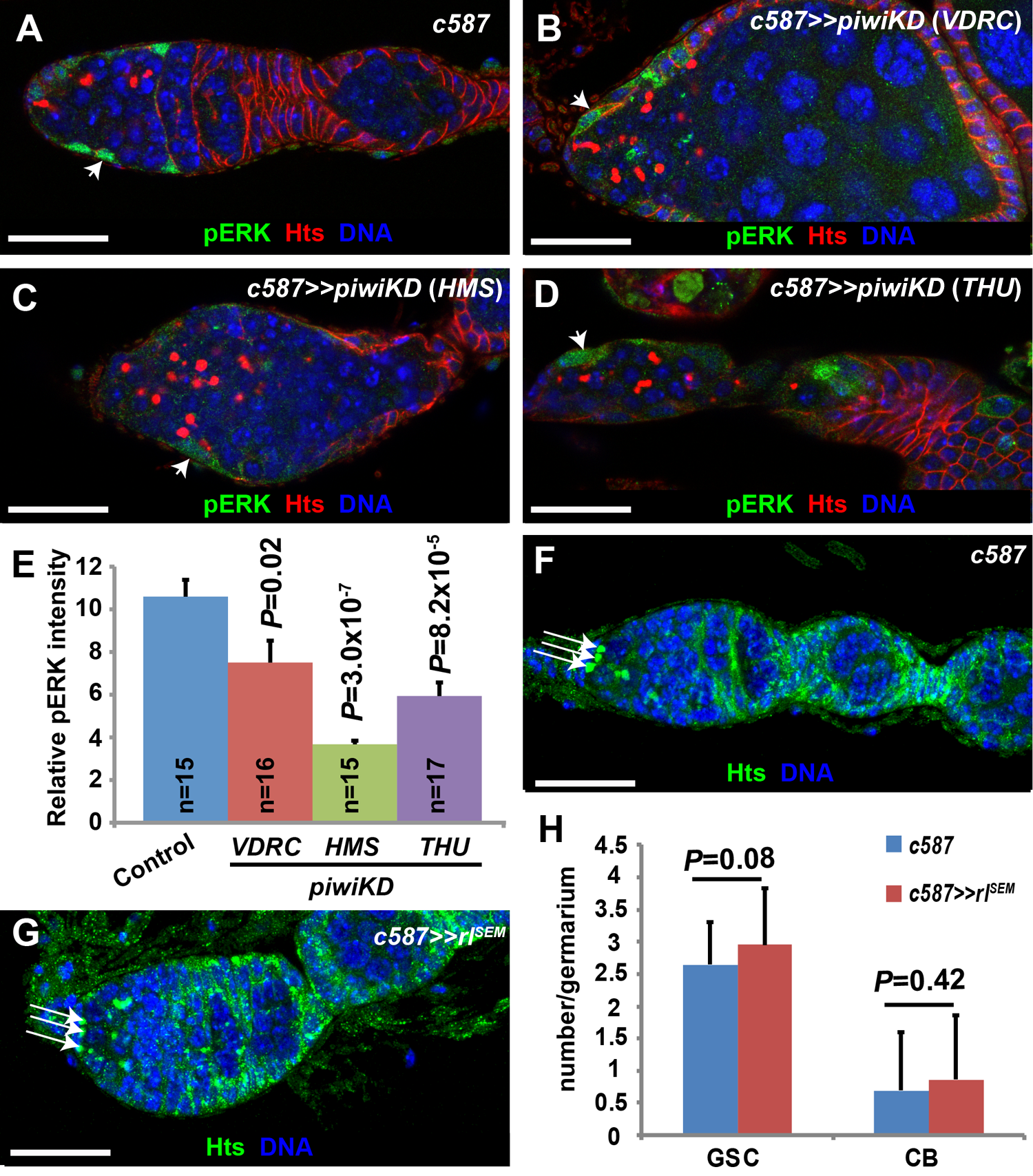

Supplement: Figure S5 — pERK activity in piwiKD ECs. (A) pERK is specifically expressed in ECs (one by arrowhead) of the control germarium. (B–E) c587-mediated piwiKD ECs (arrowheads) are often larger and show lower pERK fluorescence intensity. E shows quantification results on pERK intensity. (F–G) c587-mediated rlSEM expression does not affect GSC and CB numbers (arrows indicate GSCs). H shows that there are no significant differences in GSCs and CBs between control and rlSEM -expressing germaria. Scale bars: 25 µm. (TIF) [file pone.0090267.s005.tif]

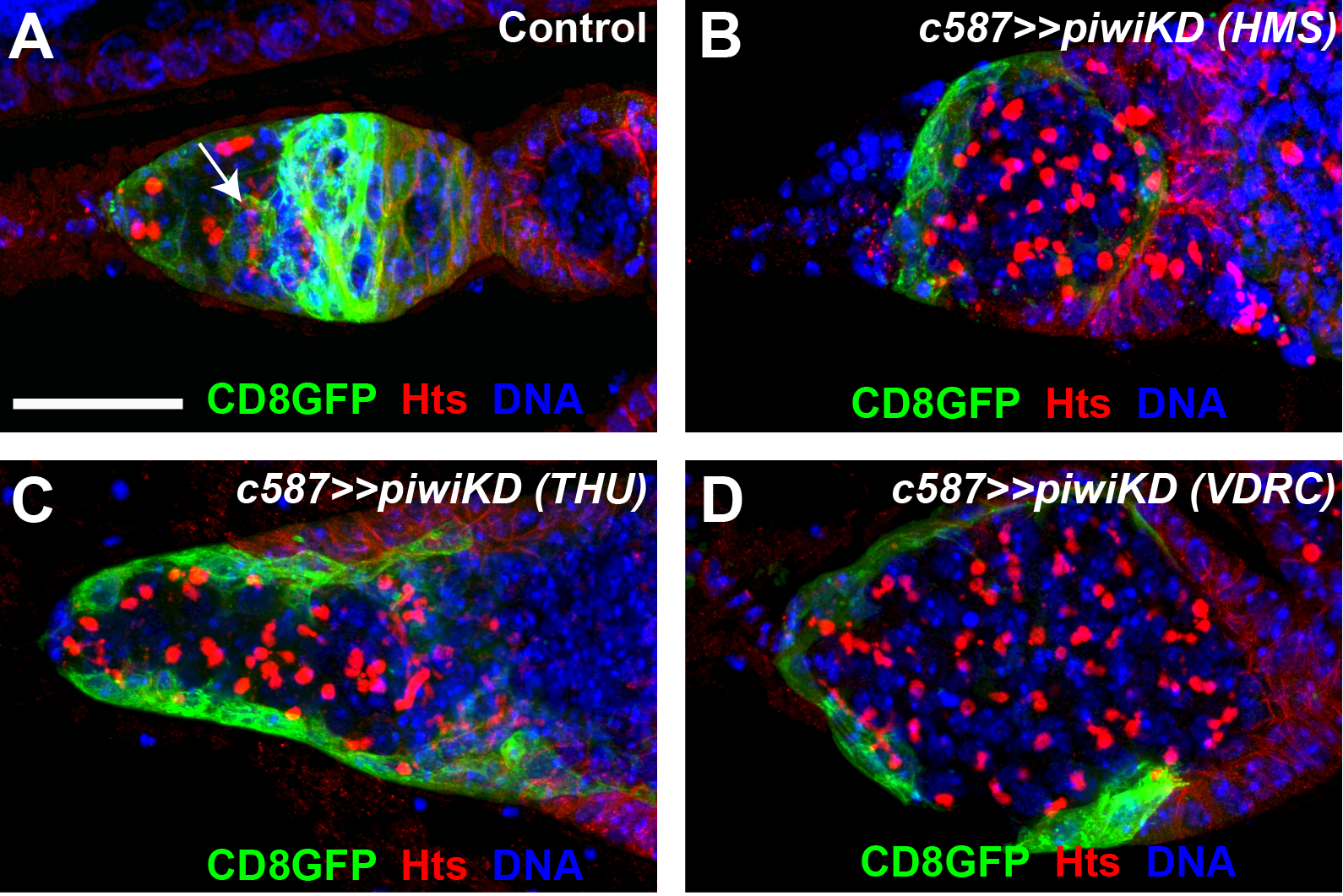

Supplement: Figure S6 — Piwi knockdown in ECs disrupts the formation of their long cellular processes. (A) c587-mediated CD8GFP expression highlights long EC cellular processes (arrows) wrapping CBs, mitotic cysts and 16-cell cysts in the control germarium. (B–D) In the c587-mediated piwiKD germaria by three RNAi lines, HMS (B), THU (C) and VDRC (D), there are no long-GFP-positive cellular processes wrapping differentiated germ cells. Scale bars: 25 µm. (TIF) [file pone.0090267.s006.tif]

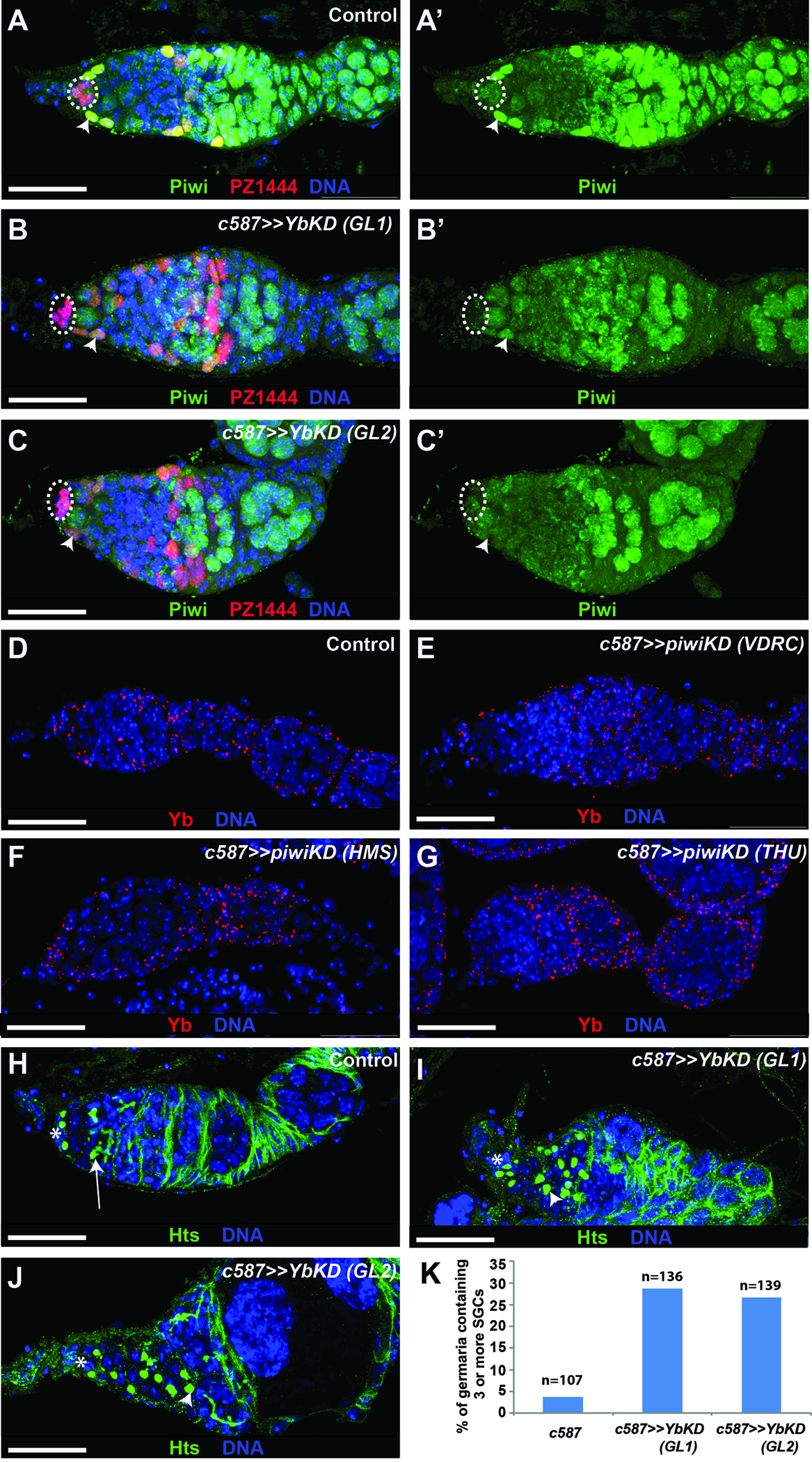

Supplement: Figure S7 — Yb is required in ECs to promote germ cell differentiation. The GSC niche is highlighted by broken lines (A–C′) or the asterisk (H–J). (A–C′) c587-mediated YbKD by two RNAi lines, GL1 (B, B′) and GL2 (C, C′), leads to a Piwi protein expression reduction in cap cells (broken lines), ECs (arrowheads) and early follicle cells in comparison with the control (A, A′). (D–G) c587-mediated piwiKD by three RNAi lines, VDRC (E), HMS (F) and THU (G), has no effect on YB protein expression in cap cells, ECs and early follicle cells in comparison with the control (D). (H) The control germarium contains three GSCs and differentiated cysts (arrow). (I–K) c587-mediated YbKD causes an accumulation of excess SGCs (arrowheads) in the germarium. K represents the quantitative results on the germaria carrying three or more SGCs. Scale bars: 25 µm. (TIF) [file pone.0090267.s007.tif]
