# Supplementary material for: Piwi Is Required in Multiple Cell Types to Control Germline Stem Cell Lineage Development in the Drosophila Ovary
Source: PLoS One. 2014 Mar 21;9(3):e90267. doi: 10.1371/journal.pone.0090267 (PMC3962343; doi:10.1371/journal.pone.0090267)
Supplement: Table S1 — This table contains the nucleotide sequences of all the primers used in this study. (DOCX) [file pone.0090267.s008.docx]

Table S1. Primers for qRT-PCR

| **Gene Name** | **Fwd Primer** | **Reverse Primer** |
| --- | --- | --- |
| *dpp* | TCGGCCAACACAGTGCGAAGTTT | TTCACGTCGAAGTGCAGCCGAAA |
| *gbb* | AATGGTTCTGCTCATGTTCGTGGC | TCAGCACTCTGTGCATGATCGTCT |
| *dally* | GAGCAACAGCAGATGCACACGAAT | GTGCACTTCAAGGGTTTCACGGTT |
| *gypsy* | ATTATCAACGAAGCCGCAGCTCAC | AATTCAGAGCCGTTGATGGTTGCC |
| *ZAM* | AACGCTCGACCTAACTAGCGGTTT | AGATCGCCAAGAACGCTGTCCATA |
| *tart* | AGAGAGGGAAAGAAGGGAAAGGGA | ATTTCCTGCCTGGTTAGATCGCCA |
| *gapdh* | AGGGAGCCACCTATGACGAAATCA | AGACGAATGGGTGTCGCTGAAGAA |
| *tbp* | TCCAGACTGGCAGCGAGAAAGTAT | AACTTGACATCGCAGGAGCCG |
| *Rpl32* | AGCGCACCAAGCACTTCATC | GACGCACTCTGTTGTCGATACC |
